# Is the right way to go in between? Rimegepant as needed provides preventive benefit. A comment on: monthly migraine days, tablet utilization, and quality of life associated with rimegepant—post hoc results from an open label safety study (BHV3000–201)

**DOI:** 10.1186/s10194-023-01565-6

**Published:** 2023-03-30

**Authors:** Andreas R. Gantenbein, Andreas Kleinschmidt

**Affiliations:** 1 Pain and Research Department, ZURZACH Care, Quellenstrasse 34, CH-5330 Bad Zurzach, Switzerland; 2 Department of Neurology, University Hospital Zurich (USZ), Zurich, Switzerland; 3 Division of Neurology, Department of Clinical Neurosciences, University Hospital Geneva (HUG) and Medical Faculty Geneva, Geneva, Switzerland

**Keywords:** As needed, Migraine, Medication overuse headache, Acute, Prophylaxis, Anti-CGRP

## Abstract

In the study by Johnston et al., gepants were meant to be taken to treat emergent migraine. It is tempting to speculate what the effect would be if patients were instructed to take a gepant as needed (PRN) or even prior to headache onset. While the latter sounds irrational at first glance, several studies have shown that a significant proportion of patients are quite proficient in predicting (or simply due to premonitory symptoms noting) their migraine attacks prior to the onset of actual headache. The study by Johnston et al. provides food for thought along these lines and should encourage us to further investigate flexible patient-controlled CGRP blocking as a third, intermediate and potentially cost-effective avenue between acute/rescue treatment and prevention/prophylaxis.

## Background

A long-standing tenet in migraine care has been the distinction of acute rescue treatment and long-term preventive treatment [1, 2]. Indeed, long-term use of acute medication harbors the risk of medication-overuse headache whereas prophylactic medication has no acute analgesic effects. There has been some evidence that continuous low-dose analgesic medication can provide prophylaxis, and combined acute and prophylactic effects from neuromodulatory stimulation have also been shown [3–6]. Yet, blocking the CGRP system seems to question the clear-cut distinction in a more fundamental way. Indeed, the onset of anti-migrainous efficacy of at least one preventive monoclonal antibody can be so rapid as to reach out into the realm of acute management [7]. Conversely, preventive properties of long-term use of acutely active anti-CGRP small molecules were recognized early on but daily application of telcagepant resulted in an increase of aminotransferase (ALAT), and the development was stopped [8]. More recently developed gepants have overcome safety concerns and atogepant and rimegepant have confirmed their prophylactic properties [9, 10]. Importantly, post hoc analysis did not show any risk for the development of medication overuse headache (MOH), when using gepants on more than 10 days per month [11, 12].

## Main text

Dose and regimen finding in drug development is still a challenge, and remain mainly based on pharmacokinetics [13]. For any prophylactic medication, however, dosage will usually be fixed at a given, maybe individually determined level. This practice is not necessarily intuitive as migraine attack frequency and hence presumably predisposition may vary largely intra-individually on various time scales [14]. One could hence conjecture that the actual need of for instance CGRP blocking should present similar variability. It would be difficult to modulate monoclonal antibody concentrations on a relevant time scale but this can readily be done for gepants. This is what the study of Johnston et al. reports [15]. Modulation of CGRP blocking was instantiated by the need for taking 75 mg of rimegepant for acute treatment, and the authors’ analysis could show that this patient-determined practice is associated with a long-term reduction in monthly migraine days that seems to be of similar effect size as the overall preventive effect of 75 mg of rimegepant taken every other day (QOD) in the pivotal study by Croop et al. [10]. Importantly, not only did headache days not increase over time with this practice but neither did gepant intake.

One possible interpretation is that in this open label study in a population suffering from frequent migraine attacks, need-driven gepant intake was simply implementing a fluctuating and putatively suboptimal preventive treatment scheme. Another interpretation discussed by the authors is that time constants of gepant action translate into the count of monthly migraine days by way of long lasting acute effects. As a third possibility, effective acute treatment may translate into better outcome, as Lipton et al. have shown [16]. Notwithstanding these interpretations, it still holds that by definition a prophylactic/preventive effect was achieved, and that this occurred with less gepant intake (and hence less cost) than in the established fixed regime of 75 mg of rimegepant every other day. Fine-grained analyses of the temporal structure of migraine attacks and associated gepant intake from the patient diaries might more clearly disentangle these different interpretations but an interesting hypothesis is that patients know best when they need CGRP blocking.

## Conclusion

In this study, gepants were meant to be taken to treat emergent migraine, at any pain intensity. It is tempting to speculate what the effect would be if patients were instructed to take a ‘prophylactic’ medication, such as a gepant, not only as needed (PRN), but even prior to headache onset. While the latter sounds irrational at first glance, several studies have shown that a significant proportion of patients are quite proficient in predicting (or simply due to premonitory symptoms noting) their migraine attacks prior to the onset of actual headache [17]. Current efforts seek to develop models of greater and better forecasting capability that could predict day-to-day need and hence guide drug intake so as to avoid actual headache [18]. The study by Johnston et al. is to our knowledge the first one to make us reconsider the distinction between acute and prophylactic medication in CGRP blocking, and might induce a paradigm change. It provides food for thought along these lines and should encourage us to further investigate need-driven patient-controlled CGRP blocking as a third, intermediate and potentially cost-effective avenue between acute/rescue treatment and prevention/prophylaxis [15].

## Data Availability

As this is a comment to the Paper (Johnston K, Harris L, Powell L, Popoff E, Coric V, L'Italien G, Schreiber CP (2022) Monthly migraine days, tablet utilization, and quality of life associated with Rimegepant—post hoc results from an open label safety study (BHV3000-201). J Headache Pain 23(1):10. doi: 10.1186/s10194-021-01378-5.) there are no own data.

## Abbreviations

ALAT: Aminotransferase
CGRP: Calcitonin gene-related peptide
MOH: Medication overuse headache
PRN: (Pro re nata) as needed
QOD: (Quaque altera die) every other day

## References

[1] Diener H-C (2019). Treatment of migraine attacks and prevention of migraine: Guidelines by the German Migraine and Headache Society and the German Society of Neurology. Clin Transl Neurosci.

[2] Ashina M (2020). Migraine. N Engl J Med.

[3] Buring JE, Peto R, Hennekens CH (1990). Low-dose aspirin for migraine prophylaxis. JAMA.

[4] Schoenen J, Jensen RH, Lanteri-Minet M, Lainez MJ, Gaul C, Goodman AM, Caparso A, May A (2013). Stimulation of the sphenopalatine ganglion (SPG) for cluster headache treatment. Pathway CH-1: a randomized, sham-controlled study. Cephalalgia.

[5] Schoenen J, Vandersmissen B, Jeangette S, Herroelen L, Vandenheede M, Gérard P, Magis D (2103). Migraine prevention with a supraorbital transcutaneous stimulator: a randomized controlled trial. Neurology.

[6] Moisset X, Pereira B, Ciampi de Andrade D, Fontaine D, Lantéri-Minet M, Mawet J (2020). Neuromodulation techniques for acute and preventive migraine treatment: a systematic review and meta-analysis of randomized controlled trials. J Headache Pain.

[7] Dodick DW, Gottschalk C, Cady R, Hirman J, Smith J, Snapinn S (2020). Eptinezumab Demonstrated Efficacy in Sustained Prevention of Episodic and Chronic Migraine Beginning on Day 1 After Dosing. Headache.

[8] Ho TW, Connor KM, Zhang Y, Pearlman E, Koppenhaver J, Fan X, Lines C, Edvinsson L, Goadsby PJ, Michelson D (2014). Randomized controlled trial of the CGRP receptor antagonist telcagepant for migraine prevention. Neurology.

[9] Goadsby PJ, Dodick DW, Ailani J, Trugman JM, Finnegan M, Lu K, Szegedi A (2020). Safety, tolerability, and efficacy of orally administered atogepant for the prevention of episodic migraine in adults: a double-blind, randomised phase 2b/3 trial. Lancet Neurol.

[10] Croop R, Lipton RB, Kudrow D, Stock DA, Kamen L, Conway CM, Stock EG, Coric V, Goadsby PJ (2021). Oral rimegepant for preventive treatment of migraine: a phase 2/3, randomised, double-blind, placebo-controlled trial. Lancet.

[11] Argyriou AA, Mantovani E, Mitsikostas DD, Vikelis M, Tamburin S (2022). A systematic review with expert opinion on the role of gepants for the preventive and abortive treatment of migraine. Expert Rev Neurother.

[12] Chan C, Goadsby PJ (2019). Recent Advances in Pharmacotherapy for Episodic Migraine. CNS Drugs.

[13] Musuamba FT (2017). Advanced Methods for Dose and Regimen Finding During Drug Development: Summary of the EMA/EFPIA Workshop on Dose Finding (London 4–5 December 2014). CPT Pharmacometrics Syst Pharmacol.

[14] Poulsen AH, Younis S, Thuraiaiyah J, Ashina M (2021). The chronobiology of migraine: a systematic review. J Headache Pain.

[15] Johnston K, Harris L, Powell L, Popoff E, Coric V, L'Italien G, Schreiber CP (2022). Monthly migraine days, tablet utilization, and quality of life associated with Rimegepant - post hoc results from an open label safety study (BHV3000-201). J Headache Pain.

[16] Lipton RB, Fanning KM, Serrano D, Reed ML, Cady R, Buse DC (2015). Ineffective acute treatment of episodic migraine is associated with new-onset chronic migraine. Neurology.

[17] Giffin NJ, Ruggiero L, Lipton RB, Silberstein SD, Tvedskov JF, Olesen J, Altman J, Goadsby PJ, Macrae A (2003). Premonitory symptoms in migraine: an electronic diary study. Neurology.

[18] Buse DC, McGinley JS, Lipton RB (2020). Predicting the Future of Migraine Attack Prediction. Headache.

